# Interleukin-33 Contributes to the Induction of Th9 Cells and Antitumor Efficacy by Dectin-1-Activated Dendritic Cells

**DOI:** 10.3389/fimmu.2018.01787

**Published:** 2018-07-31

**Authors:** Jintong Chen, Yinghua Zhao, Yuxue Jiang, Sujun Gao, Yiming Wang, Dongjiao Wang, Alison Wang, Huanfa Yi, Rui Gu, Qing Yi, Siqing Wang

**Affiliations:** ^1^Department of Cancer Immunology, The First Hospital of Jilin University, Changchun, China; ^2^Department of Hematology, The First Hospital of Jilin University, Changchun, China; ^3^Department of Orthopedics, China-Japan Union Hospital of Jilin University, Changchun, China; ^4^The First Hospital and Institute of Immunology, Jilin University, Changchun, China; ^5^Department of Cancer Biology, Lerner Research Institute, Cleveland Clinic, Cleveland, OH, United States

**Keywords:** dendritic cells, dectin-1, Th9, interleukin-33, cancer immunotherapy

## Abstract

We recently discovered that dectin-1-activated dendritic cells (DCs) drive potent T helper (Th) 9 cell responses and antitumor immunity. However, the underlying mechanisms need to be further defined. The cytokine microenvironment is critical for Th cell differentiation. Here, we show that dectin-1 activation enhances interleukin (IL)-33 expression in DCs. We found that blocking IL-33/ST2 inhibits dectin-1-activated DC-induced Th9 cell differentiation. More importantly, the addition of IL-33 further promotes Th9 cell priming and antitumor efficacy induced by dectin-1-activated DCs. Mechanistically, in addition to the promotion of Th9 and Th1 cells, dectin-1-activated DCs combined with IL-33 abolish the activity of IL-33 in the induction of regulatory T cells. Furthermore, the combined treatment of dectin-1-activated DCs and IL-33 increases the frequencies of CD4^+^ T cells by fostering their proliferation and inhibiting their exhaustive differentiation. Thus, our results demonstrate the important role of IL-33 in dectin-1-activated DC-induced Th9 cell differentiation and antitumor efficacy, and suggest that the combination of dectin-1-activated DCs and IL-33 may present a new effective modality of DC-based vaccines in tumor immunotherapy.

## Introduction

Dendritic cell (DC) vaccines offer a great promise for tumor therapy; however, clinical trials of DC-based tumor therapy have shown only limited clinical benefits (1, 2). There is an urgent need for further investigation of more effective DC vaccines for tumor therapy. DCs exert their antitumor effects mainly through the induction of antitumor effector T cells (3, 4). Naive CD4^+^ T cells can differentiate into various T helper (Th) cell subsets with different cytokine-production profiles and effector functions (5). Th9 cells are a new Th cell subset that produces interleukin 9 (IL-9) (6, 7). Th9 cells and IL-9 are pro-inflammatory, shown to be implicated in allergy, autoimmunity, and infectious diseases (8, 9). We and others recently found that tumor-specific Th9 cells induce potent antitumor efficacy in mouse tumor models, better than other Th cells (10–12). Mechanisms underlying Th9-induced antitumor immunity are not fully defined. Th9 cells may exert their antitumor effects through the activation of other effectors, such as tumor-specific CD8^+^ CTLs and mast cells (10, 11). Th9 cells may also exert a direct antitumor activity at the tumor site (12, 13). These findings suggest that strategies to preferably stimulate DC induction of Th9 cells may greatly improve the antitumor efficacy of DC-based tumor therapy.

The cytokine milieu is the most important determinant of the fate of T cell differentiation (5). Th9 cells can be generated *in vitro* by culturing naïve CD4^+^ T cell with IL-4 and transforming growth factor β (TGF-β) (6, 7). However, other cytokines, such as TL1A, OX40L, and IL-25, can also promote Th9 cell development (14–16). In addition, multiple transcription factors, such as PU.1, IRF4, and Foxo1, are shown to be involved in Th9 cell differentiation (17–19). Dectin-1 is a member of C-type lectin-like receptors that plays an important role in anti-fungal immune responses (20–22). We recently found that DCs activated *via* dectin-1 trigger potent antitumor effects through the induction of Th9 cells (12, 13). We further found that dectin-1 stimulates DCs to overexpress 42 cytokines and costimulatory molecules (12), and among these, TL1A and OX40L were proved to contribute to dectin-1-activated DC-induced Th9 cell priming (12). However, the role of the other cytokines in dectin-1-activated DC-induced Th9 cell differentiation and antitumor efficacy remains unclear.

*Il33*, the gene encoding IL-33, is the third highest expressed gene among the 42 upregulated cytokine and costimulatory molecule genes in dectin-1-activated DCs (12). IL-33 is a member of the IL-1 superfamily cytokines and a ligand for IL-1 receptor-like 1 (IL1RL1, also known as ST2) (23, 24). IL-33 has been reported to be implicated in multiple allergic disorders by stimulating Th2-associated cell responses (25). In cancer immunology, IL-33 inhibits tumor growth through activation of CD8^+^ T and NK cells (26, 27). IL-33 enhances the intratumor accumulation of type 2 innate lymphoid cells and eosinophils, which mediate potent antitumor activity (28, 29). IL-33 can also promote the antitumor immunity by restoring the activation and maturation of DCs and inhibiting the differentiation and function of granulocytic myeloid-derived suppressor cells in tumors (30, 31). IL-33 stimulates DC expression of OX40L (25), which may favor the induction of Th9 cells (12, 15). Recently, Ramadan et al. showed that IL-33-treated IL-9-producing T (T9) cells have more potent antileukemic capacity than untreated T9 cells (32). Based on these observations, we hypothesized that IL-33 would greatly contribute to dectin-1-activated DC-induced Th9 cell differentiation and antitumor efficacy.

In this study, we showed that dectin-1 activation stimulates IL-33 expression in DCs. We found that IL-33 contributes to dectin-1-activated DC-induced Th9 cell differentiation. More importantly, the addition of IL-33 further promotes dectin-1-activated DC induction of Th9 cells and antitumor efficacy. Our results demonstrate the important role of IL-33 in dectin-1-activated DC-induced antitumor immunity and may have important clinical implications.

## Materials and Methods

### Mice and Cell Lines

C57BL/6, OT-II [C57BL/6^−^ Tg(TcraTcrb)425Cbn/J], and Balb/c mice were purchased from the Jackson Laboratory. Phenotype of OT-II transgenic mice was examined and confirmed (Figures S1A,B in Supplementary Material). Dectin-1^−/−^ mice were provided by G. Brown (University of Aberdeen, Aberdeen, Scotland). All mice were housed and bred under specific pathogen-free conditions at Animal Center of The First Hospital of Jilin University. Mice were used for experiments at age 6–8 weeks. All animal studies were conducted according to the ethical guidelines of the Animal Ethical Committee of First Hospital of Jilin University.

B16, B16-OVA, and MPC-11 were purchased from ATCC (Rockville, MD, USA). Cells were cultured in RPMI 1640 medium supplemented with 10% heat-inactivated fetal bovine serum (FBS, Hyclone), 100 U/mL penicillin (Hyclone), and 100 mg/mL streptomycin (Hyclone). Cells were grown in standard (37°C, 5% CO_2_) culture incubators.

### Reagents and Antibodies (Abs)

Recombinant mouse GM-CSF, TNF-α, IL-1β, and IL-4 were purchased from Peprotech. Recombinant mouse IL-33 and human TGF-β were purchased from R&D Systems. OVA (323–339, ISQ AVH AAH AEI NEA GR) peptide used in OT-II mouse model and TRP-1 (106–130, SGH NCG TCR PGW RGA ACN QKI LTV R) peptide were purchased from GL Biochem (Shanghai) Ltd. Functional anti-mouse CD3e and CD28 antibodies (mAbs) were purchased from eBioscience. ST2-blocking mAb (αST2), IL-33 neutralization mAb (αIL33), IL-9 neutralization mAb (αIL9), and control IgG were purchased from R&D Systems. Curdlan and Scleroglucan were purchased from Sigma-Aldrich and Invivogen, respectively.

### Generation of DCs

Dendritic cells were generated as described previously (12). In brief, bone marrow cells were cultured in RPMI 1640 complete medium supplemented with GM-CSF (20 ng/mL) and IL-4 (10 ng/mL). At day 4, the culture medium was replaced with fresh GM-CSF (10 ng/mL) and IL-4 (10 ng/mL)-containing medium. At day 7, the semi-adherent cells were collected as immature DCs (iDCs) and matured by TNF-α (10 ng/mL) and IL-1β (10 ng/mL) (BMDCs), Curdlan (5 µg/mL, CurDCs) or Scleroglucan (10 µg/mL, SclDCs) for 48 h. At day 9, the semi-adherent cells were collected as mature DCs (mDCs) for further experiments.

In some experiments, iDCs were generated from wild-type (WT) and Dectin-1^−/−^ C57BL/6 mice and matured with TNF-α/IL-1β (BMDCs), Curdlan (CurDCs), or Scleroglucan (SclDCs) for 2 days. mDCs were analyzed for IL-33 gene expression by flow cytometry, quantitative polymerase chain reaction (qPCR), and enzyme-linked immunosorbent assay (ELISA).

### Gene Expression Profiling Data

Microarray analyses of the gene expression profiles of BMDCs and CurDCs were performed previously (12). Data are stored in the GEO repository and is accessible under the accession number GSE81111.

### Flow Cytometry Analysis

Immunofluorescence surface staining was performed as previously described (12). The fluorescence-labeled mAbs against CD3, CD4, CD25, CD62L, and PD-1 were purchased from BD Biosciences; APC-labeled ST2 Ab was purchased from BioLegend. PE labeled anti-mouse TCR Vβ5.1, 5.2 (139504, clone MR9-4) was purchased from BioLegend.

Intracellular staining was performed as previously described (12). PE- or Pacific Blue-conjugated mAbs against IL-9, IFN-γ, IL-17, GzmB, Foxp3, and Ki67 were purchased from BioLegend. After staining, cell samples were analyzed by a BD LSRFortessa™ cytometer.

### *In Vitro* Th9 Cell Differentiation

Naïve CD4^+^ T cells (CD4^+^CD25^−^CD62L^hi^) were purified by fluorescence activated cell sorter (FACS) from mouse spleens and cocultured at 1 × 10^5^ per well with BMDCs or CurDCs (1 × 10^5^/well) in the presence of plate-bound anti-CD3 (2 µg/mL) plus TGF-β (3 ng/mL) and IL-4 (10 ng/mL). Cells from cultures without addition of TGF-β and IL-4 were used as Th0 cells. In some cell cultures, αST2 (5 µg/mL) or IL-33 (50 ng/mL) were added. After 3 days of culture, cells were harvested and analyzed by flow cytometry and/or qPCR.

### *In Vitro* Regulatory T (Treg) Cell Differentiation

Naïve CD4^+^ T cells were isolated from mouse spleens by FACS and cocultured at 1 × 10^5^ per well with BMDCs or CurDCs (1 × 10^5^/well) in the presence of plate-bound anti-CD3 (2 µg/mL) and soluble anti-CD28 (2 µg/mL) plus TGF-β (3 ng/mL). In some cell cultures, IL-33 (50 ng/mL) was added. After 3 days of culture, cells were harvested and analyzed by qPCR.

### Quantitative Polymerase Chain Reaction

Total RNA was extracted from cells using an EasyPure RNA Kit (TransGen Biotech), and cDNA was synthesized with an All-in-One First-Strand cDNA Synthesis SuperMix (TransGen). The mRNA levels of *Foxp3, Ifng, Il2, Il9, Il17, Il33, Irf4, Gzmb, Pdcd1, Prf1*, and *St2* (gene for the transmembrane form, ST2L) by DCs or Th cells were analyzed. Expression was normalized to the expression of the housekeeping gene *Gapdh*. Primer sets for *Foxp3, Ifng, Il9, Il17*, and *Irf4* were shown in the previous publication (12). Primer sets for *Il2, Il33, Gzmb, Pdcd1, Prf1*, and *St2* are listed in Table S1 in Supplementary Material.

### Enzyme-Linked Immunosorbent Assay

Concentrations of IL-33, IL-9, and IFN-γ in culture supernatants were detected by ELISAs as previously described (12). IL-33 capture/detection Abs were purchased from R&D Systems. Recombinant mouse IL-33 (aa109–266) (ELISA standard) was purchased from R&D Systems. Capture/detection Abs for IL-9 and IFN-γ were purchased from BD Biosciences. Recombinant mouse IL-9 and IFN-γ used as the standards in ELISAs were purchased from R&D Systems and BD Biosciences, respectively. Avidin-HRP was purchased from BioLegend.

### Tumor Immunotherapy Experiments

BMDCs and CurDCs were pulsed with OT-II OVA peptides (5 µg/mL) for 2–4 h and then harvested for mouse immunization (*n* = 4–5/group). B16-OVA (2 × 10^5^ cells/mouse) were injected subcutaneously into OT-II mice. On day 3 after tumor challenge, mice were randomly divided into groups and given two weekly subcutaneous immunizations with treated DCs (1 × 10^6^ cells/mouse). Mice injected with PBS served as controls. In some experimental groups, mice were given IL-33 (250 ng/mouse) every 3 days starting at 1 day after the first DC immunization. Tumor volume was calculated by the formula: 3.14 × (mean diameter)^3^/6. Mice were killed when the tumor diameter reached to the range between 1.5 and 2 cm.

We also used MPC-11 myeloma tumor model to further examine the role of dectin-1-activated DCs plus IL-33 in tumor therapy. MPC-11 tumor cells (1 × 10^6^ cells/mouse) were injected subcutaneously into Balb/c mice. MPC-11 tumor cell lysates were generated by five rapid freeze-thaw cycles as described previously (33) and were used as tumor antigens. On day 3 after tumor challenge, BMDCs or CurDCs (1 × 10^6^ cells/mouse) loaded with tumor cell lysates (100 μg/1 × 10^6^ DCs) were used for the tumor treatment in the presence or absence of IL-33. Mice were treated twice (1 week apart). Mice injected with PBS served as controls. Tumor growth was monitored overtime.

### *In Vivo* Functional Tests of IL-33/ST2 in DC-Induced T Cell Differentiation

BMDCs and CurDCs were pulsed with OT-II OVA peptides (5 µg/mL). Mice were given two weekly subcutaneous immunizations with 1 × 10^6^ treated DCs. Mice injected with PBS served as controls. In some experiments, mice were given control IgG or blocking anti-ST2 mAb (αST2, 25 μg/mouse) or IL-33 (250 ng/mouse) every 3 days starting at 1 day after the first DC immunization. On day 3 after the second DC immunization, total leukocytes from spleens and lymph nodes were restimulated with peptide-pulsed DCs for 24 h. Cells from PBS control mice were cultured without addition of DCs. Culture cells and supernatants were collected and analyzed by qPCR, ELISA, and flow cytometry.

### Statistical Analysis

The Student’s *t*-test (2 groups) and one-way ANOVA (≥3 groups) were used to compare various experimental groups. A *P* value of less than 0.05 was considered significant.

## Results

### Dectin-1 Signaling Increases IL-33 Expression in DCs

**Figure 1:**
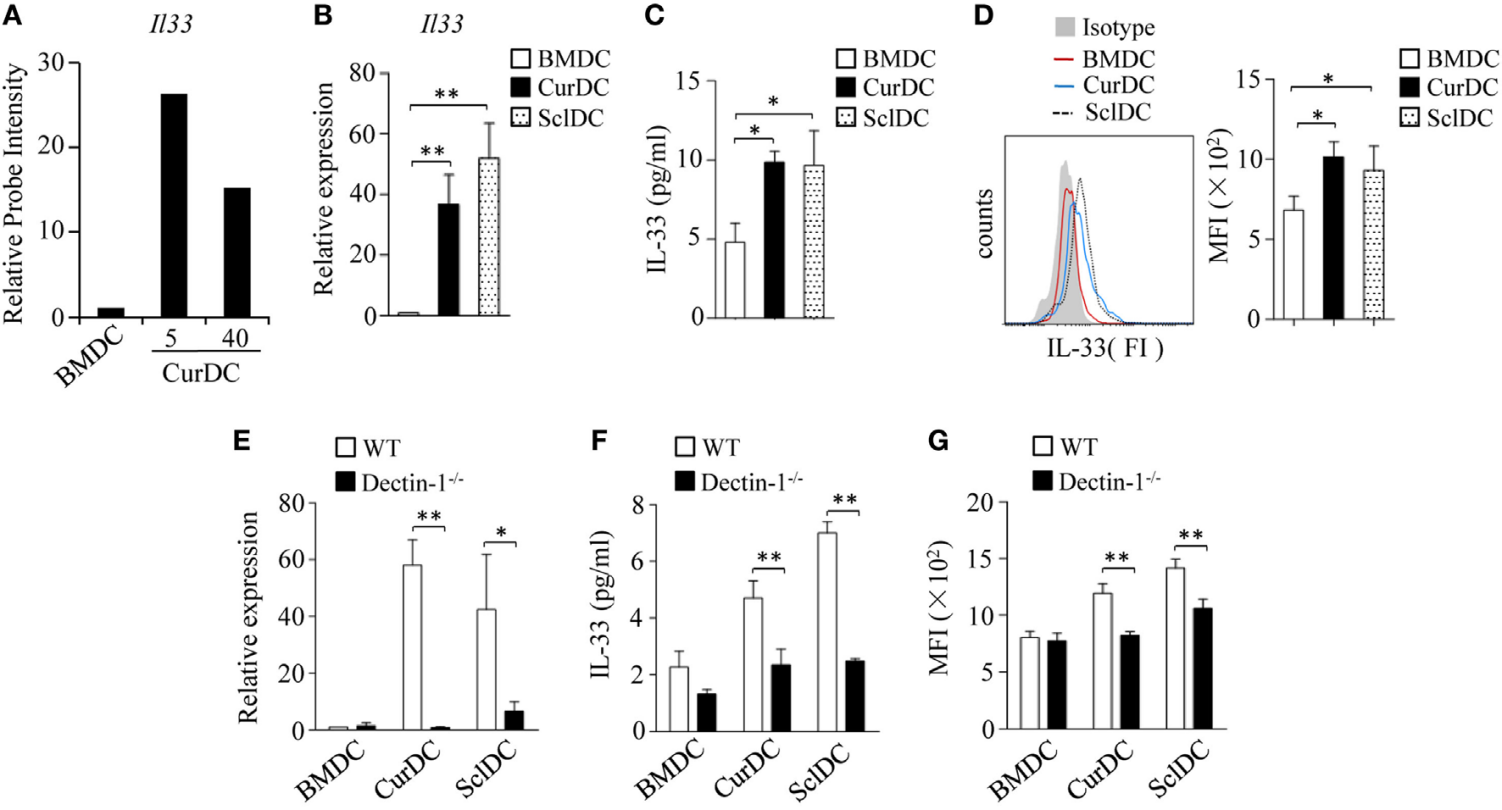
Dectin-1 signaling stimulates interleukin (IL)-33 expression in dendritic cells (DCs). **(A)** Relative abundance of *Il33* mRNA levels in DCs extracted from microarray gene expression data. Mouse DCs matured by TNF-α/IL-1β (BMDC), or Curdlan (CurDC) at doses of 5 and 40 µg/mL for 48 h were analyzed by microarrays. The probe intensity of *Il33* in BMDCs was set at 1. **(B–D)** Mouse immature DCs were matured by TNF-α/IL-1β, Curdlan, or Scleroglucan (SclDC) for 48 h. **(B)** Quantitative polymerase chain reaction (qPCR) analyzed the mRNA levels of *Il33* in DCs. The experiments were performed three times (*n* = 3). **(C)** Enzyme-linked immunosorbent assay (ELISA) assessed IL-33 secretion by DCs. The experiments were performed three times (*n* = 3). **(D)** Intracellular staining of IL-33 in DCs. Right, summarized results of three independent experiments obtained as the left. MFI, mean fluorescence intensity. The experiments were performed three times (*n* = 3). **(E–G)** BMDCs, CurDCs, and SclDCs were generated from wild-type (WT) or dectin-1^−/−^ mice. Same as in **(B–D)**, the mRNA **(E)** and protein **(F,G)** levels of IL-33 expressed by DCs were analyzed by qPCR, ELISA, and intracellular staining separately. The experiments were performed three times (*n* = 3). Results shown are the mean ± SD of three **(B–G)** independent experiments. *P < 0.05; **P < 0.01.

We first examined the role of dectin-1 activation in IL-33 expression in DCs. Mouse iDCs were matured with TNF-α plus IL-1β (BMDCs) or a selective dectin-1 agonist Curdlan (CurDCs) at dosages of 5 and 40 µg/mL. Microarray analysis detected increased IL-33 expression in CurDCs compared to BMDCs (Figure 1A). The increased expression of IL-33 by CurDCs compared to BMDCs was confirmed by qPCR, ELISA, and Flowcytometry (Figures 1B–D). We also used another dectin-1 agonist, Scleroglucan, to mature mouse DCs (SclDCs). Similarly, SclDCs had higher expression of IL-33 than BMDCs (Figures 1B–D). These results indicated that dectin-1 agonists drove IL-33 expression in DCs.

To further confirm the role of dectin-1 activation in IL-33 expression in DCs, we generated CurDCs and SclDCs from WT and dectin-1 knockout (dectin-1^−/−^) mice. Dectin-1-deficiency largely abolished the increase of IL-33 expression induced by Curdlan and Scleroglucan in DCs (Figures 1E–G). Notably, CurDCs and BMDCs had comparable expression of IL-33 receptor ST2 (Figures S2A,B in Supplementary Material). Together, these results demonstrated the role of dectin-1 activation in stimulating IL-33 expression in DCs.

### IL-33 Contributes to Dectin-1-Activated DC-Induced Th9 Differentiation

**Figure 2:**
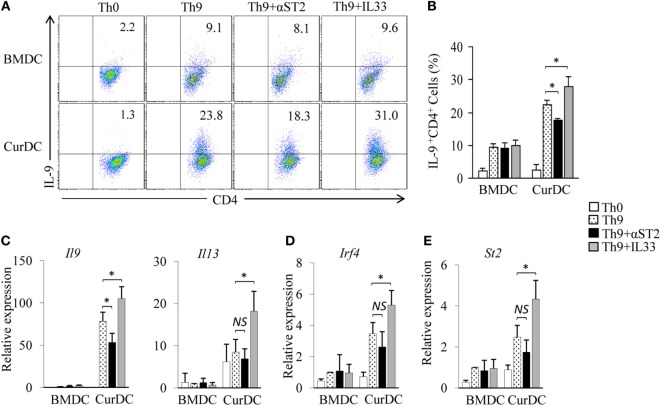
Interleukin (IL)-33 contributes to dectin-1-activated dendritic cell (DC)-induced Th9 priming. Naïve CD4^+^ T cells were cocultured with BMDCs or CurDCs under Th9-polarizing conditions with or without addition of an anti-ST2 blocking antibody (αST2) or IL-33. Cell cultures without (Th0) addition of Th9-polarizing cytokines transforming growth factor β and IL-4 were used as controls. Cells were cultured for 3 days. **(A)** Flow cytometry analysis of IL-9-expressing CD4^+^ T cells. Numbers in the dot plots represent the percentages of IL-9^+^CD4^+^ T cells. **(B)** Summarized results of three independent experiments obtained in **(A)**. The experiments were performed three times (*n* = 3). Quantitative polymerase chain reaction analysis of *Il9* and *Il13* **(C)**, *Irf4* **(D)**, and *St2* **(E)** in T cells. Expression was normalized to *Gapdh* and set at 1 in BMDC-induced Th9 cells. The experiments were performed three times (*n* = 3). Data are representative of three **(A)** independent experiments or presented as mean ± SD of three **(B–E)** independent experiments. *NS*, non-significant; *P < 0.05.

Dectin-1-activated DCs potently promote Th9 cell differentiation *in vitro* and *in vivo* (12). To determine the role of IL-33 in dectin-1-activated DC-induced Th9 cell differentiation, a blocking antibody against ST2 (αST2) was used. Naïve CD4^+^ T cells were cocultured with CurDCs under Th9-polarizing conditions with or without the addition of αST2. αST2 treatment moderately decreased CurDC-induced Th9 cell development (Figures 2A,B) and IL-9 expression (Figures 2A–C) and slightly inhibited the expression of Th9-related transcription factor *Irf4* (Figure 2D) in CurDC-induced Th9 cells as compared to untreated controls; whereas αST2 treatment did not affect BMDC-induced Th9 cell development (Figures 2A,B) and *Il9* (Figure 2C) and *Irf4* (Figure 2D) expression in BMDC-induced Th9 cells. Furthermore, αST2 treatment did not alter *Il13* expression in CurDC-induced Th9 cells (Figure 2C). These results indicated that IL-33 partially mediates dectin-1-activated DC-induced Th9 cell differentiation.

To further explore the effects of IL-33 on dectin-1-activated DC-induced Th9 cell differentiation, cytokine IL-33 was added during Th9 induction by CurDCs. The addition of IL-33 further stimulated CurDC-induced Th9 cell development (Figures 2A,B) and increased IL-9 (Figures 2A–C), *Il13* (Figure 2C), and *Irf4* (Figure 2D) expression in Th9 cells primed by CurDCs. However, the addition of IL-33 failed to enhance BMDC-induced Th9 cell differentiation, as demonstrated by the comparable expression levels of IL-9 (Figures 2A–C) and *Irf4* (Figure 2D) in Th9 cells primed by BMDCs with or without addition of IL-33. Interestingly, the addition of IL-33 had minor effects on ST2 expression by BMDCs or CurDCs (Figures S2 and S3 in Supplementary Material). Collectively, these results demonstrated the contribution of IL-33 to dectin-1-activated DC-induced Th9 cell differentiation.

### IL-33 Promotes Dectin-1-Activated DC-Induced Th9/1 Cells *In Vivo*

**Figure 3:**
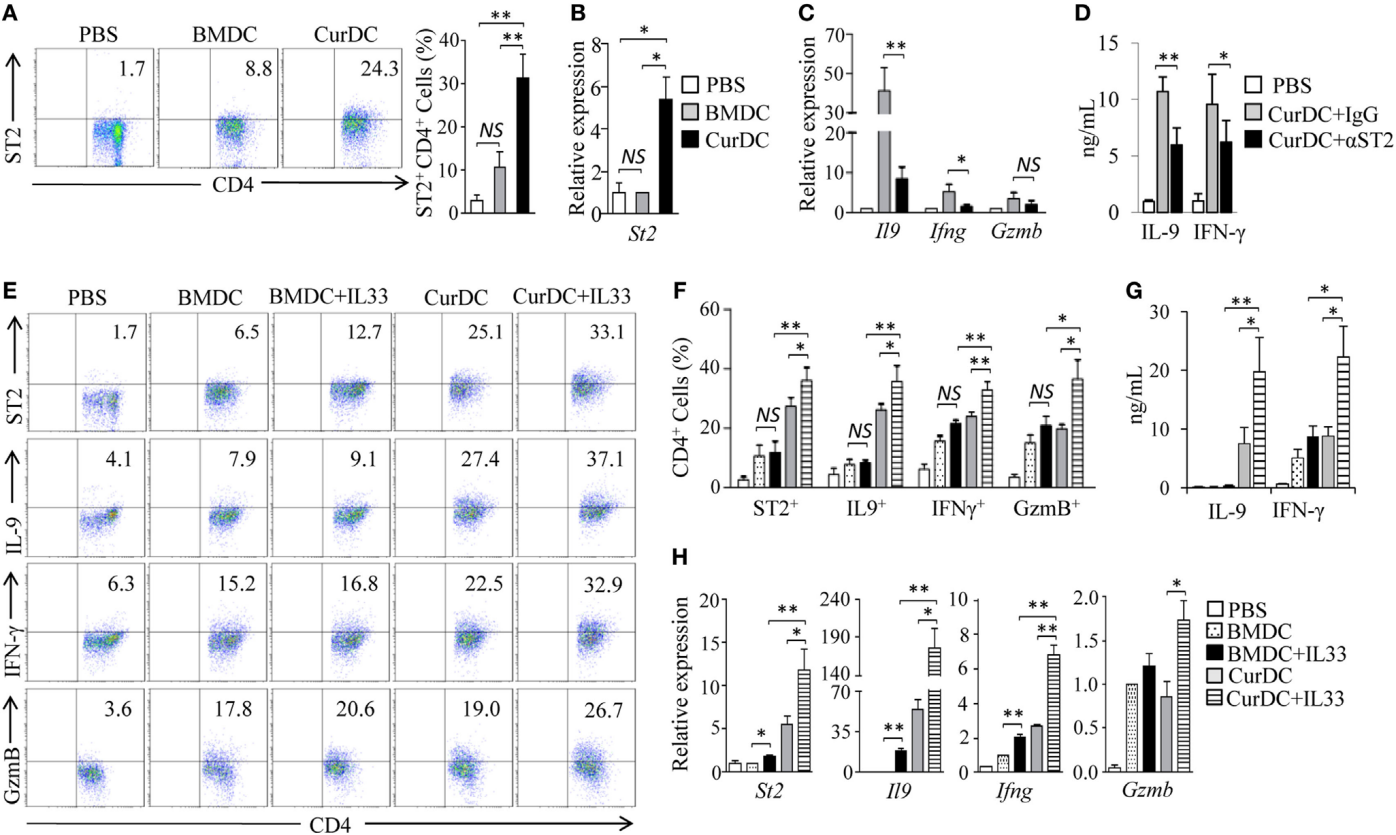
Interleukin (IL)-33/ST2 axis contributes to dectin-1-activated dendritic cell (DC)-induced Th9 priming *in vivo*. **(A,B)** OT-II mice (two to three mice/group) were immunized twice (1 week apart) with OVA-peptide-pulsed BMDCs or CurDCs. PBS served as a control. On day 3 after the second immunization, spleen cells were harvested and restimulated separately with OVA-peptide-pulsed BMDCs and CurDCs for 2 days in the culture. **(A)** Flow cytometry of ST2-expressing CD4^+^ T cells. Numbers in the dot plots represent the percentages of ST2^+^CD4^+^ T cells. Right, summarized results of three independent experiments obtained as at left. The experiments were performed three times (*n* = 3). **(B)** Quantitative polymerase chain reaction (qPCR) analysis of *St2* in CD4^+^ T cells. The experiments were performed three times (*n* = 3). **(C,D)** OT-II mice (two to three mice/group) were immunized twice (1 week apart) with OVA-peptide-pulsed CurDCs in the presence of control IgG or αST2. PBS served as a control. On day 3 after the second immunization, spleen cells were restimulated separately with OVA-peptide-pulsed CurDCs plus IgG or αST2 for 48 h. **(C)** qPCR analyses of *Il9, Ifng*, and *Gzmb* in CD4^+^ T cells. The experiments were performed three times (*n* = 3). **(D)** Enzyme-linked immunosorbent assay (ELISA) assessed IL-9 and IFN-γ in the cultures. The experiments were performed three times (*n* = 3). **(E–H)** OT-II mice (two to three mice/group) were immunized twice (1 week apart) with OVA-peptide-pulsed BMDCs or CurDCs. In addition, some of the mice immunized with BMDCs or CurDCs were given IL-33 (250 ng/mouse) every 3 days, beginning on the day of the first immunization. Mice received PBS served as controls. Mouse spleen cells were restimulated with BMDCs or CurDCs in the presence or absence of IL-33 (50 ng/mL) for 2 days in the culture. **(E)** Flow cytometry of ST2-, IFN-γ-, IL-9-, or GzmB-producing CD4^+^ T cells. Numbers in the dot plots represent the percentages of double-positive T helper cells. **(F)** Summarized results of three independent experiments obtained in **(A)**. The experiments were performed three times (*n* = 3). **(G)** ELISA assays of IL-9 and IFN-γ in the culture. The experiments were performed three times (*n* = 3). **(H)** qPCR analyses of *St2, Il9, Ifng*, and *Gzmb* in CD4^+^ T cells. The experiments were performed three times (*n* = 3). Data are representative of three **(A,E)** independent experiments or presented as mean ± SD of three **(A–D,F–H)** independent experiments. *NS*, non-significant; *P < 0.05; **P < 0.01.

To examined the effects of dectin-1-activated DC vaccination on ST2 expression in Th cells. OT-II mice were immunized by OVA-peptide-pulsed BMDCs and CurDCs. CurDC-treated mice had higher percentages of ST2-expressing CD4^+^ (ST2^+^CD4^+^) T cells in spleen cells (Figure 3A) and higher levels of *St2* mRNA expression in CD4^+^ T cells (Figure 3B) than mice treated with BMDCs or PBS controls. However, there was no difference in ST2 expression by CD4^+^ T cells from mice immunized with BMDCs compared to PBS controls (Figures 3A,B). In addition, the immunization of CurDCs compared to BMDCs or PBS control also led to higher frequencies of ST2^+^CD4^+^ T cells in C57BL/6 mouse model (Figures S4A,B in Supplementary Material). These results demonstrated that dectin-1-activated DCs stimulated CD4^+^ T cell expression of ST2 *in vivo*.

Dectin-1-activated DC vaccination promoted the development of Th9, Th1, and GzmB-expressing Th cells (Figures S5A–E in Supplementary Material) (12). To determine whether IL-33 contributes to the production of the Th cells *in vivo*, αST2 was used during CurDC immunization. A blockade of IL-33/ST2 with αST2 partially inhibited dectin-1-activated DC-induced Th9 and Th1 cell differentiation, as demonstrated by the lower expression levels of IL-9 and IFN-γ in T cell from mice immunized with CurDCs plus αST2 compared to CurDCs alone (Figures 3C,D). Together, these data demonstrated that IL-33/ST2 axis contributed to dectin-1-activated DC induction of Th9 and Th1 cells *in vivo*.

We next examined the effects of IL-33 on the induction of Th9 and Th1 cells *in vivo*. OT-II mice were immunized with OVA peptide-pulsed BMDCs or CurDCs with or without addition of IL-33. While no increase of IL-9^+^CD4^+^ (Th9), IFN-γ^+^CD4^+^ (Th1), and GzmB-expressing CD4^+^ (GzmB^+^CD4^+^) T cells were observed in mice immunized with BMDCs plus IL-33 compared to BMDCs alone (Figures 3E,F), mice immunized with CurDCs plus IL-33 had higher frequencies of Th9, Th1, and GzmB^+^CD4^+^ T cells than these receiving CurDCs or BMDCs plus IL-33 (Figures 3E,F). ELISA and/or qPCR further confirmed the increased expression of IL-9, IFN-γ, and *Gzmb* in mice immunized with CurDCs plus IL-33 compared with CurDCs or BMDCs plus IL-33 (Figures 3G,H). Interestingly, Th cells treated with CurDCs plus IL-33 expressed higher levels of ST2 than these treated with CurDCs or BMDCs plus IL-33 both *in vivo* (Figures 3E,F,H) and *in vitro* (Figure 2E).

Lymphocyte infiltration in tumors plays a central role in mediating the antitumor effects induced by DC-based tumor therapy. We next analyzed the tumor-infiltrating lymphocytes (TILs) in B16-OVA tumor-bearing OT-II mice immunized with CurDCs plus IL-33. As shown in Figures S6A,B in Supplementary Material, mice immunized with CurDCs plus IL-33 had higher frequencies of IL-9^+^CD4^+^, IFN-γ^+^CD4^+^, and GzmB^+^CD4^+^ TILs than mice immunized with CurDCs alone or BMDCs plus IL-33, whereas mice immunized with BMDCs plus IL-33 had similar frequencies of IL-9^+^CD4^+^, IFN-γ^+^CD4^+^, and GzmB^+^CD4^+^ TILs as compared to those immunized by BMDCs alone (Figures S6A,B in Supplementary Material). Together, these results demonstrated that IL-33 stimulated dectin-1-activated DCs to induce Th9 and Th1 cells *in vivo*, suggesting the potential of IL-33 in promoting dectin-1-activated DC-induced antitumor efficacy.

### IL-33 Promotes the Antitumor Efficacy of Dectin-1-Activated DCs

**Figure 4:**
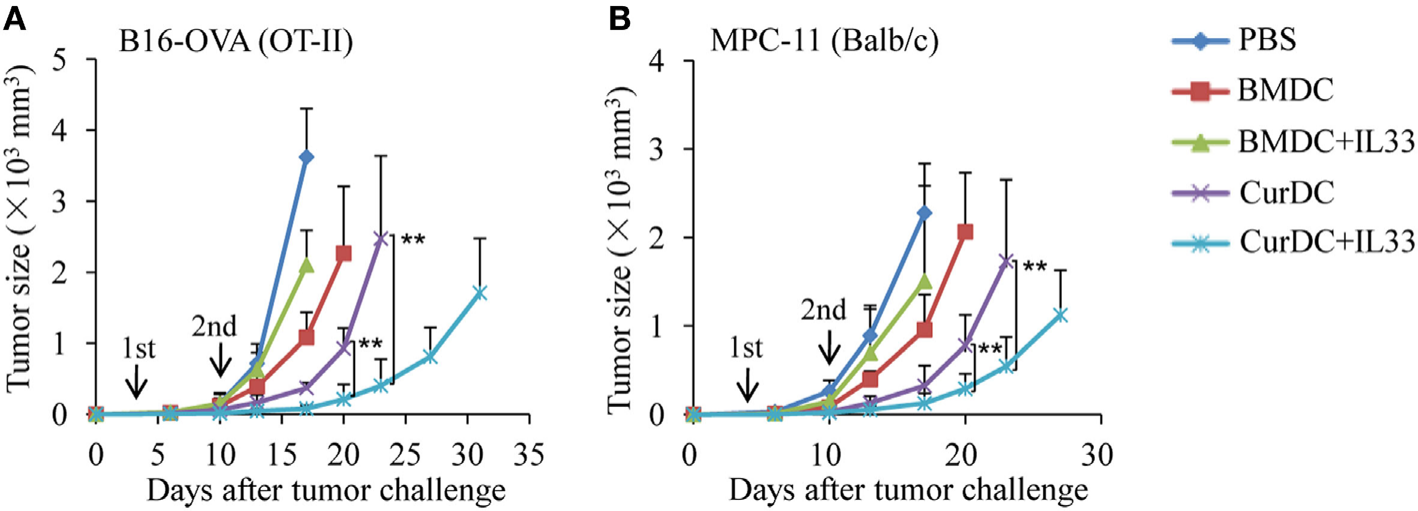
Interleukin (IL)-33 promotes dectin-1-activated dendritic cell (DC)-induced antitumor efficacy *in vivo*. **(A)** OT-II mice were injected subcutaneously with 1 × 10^5^ B16-OVA cells. On day 3 after tumor challenge, mice (five mice/group) were given two weekly subcutaneously immunizations with 1 × 10^6^ OVA-peptide-pulsed BMDCs or CurDCs. In addition, some of the mice immunized with BMDCs or CurDCs were given IL-33 (250 ng/mouse) every 3 days, beginning on the day of the first immunization. Mice received PBS served as controls. Shown are the tumor growth curves. The experiments were performed twice with a total of 10 mice per group (*n* = 10). **(B)** Balb/c mice were injected subcutaneously with 1 × 10^6^ MPC-11 cells. On day 3 after tumor challenge, mice (five mice/group) were given two weekly subcutaneously immunizations with 1 × 10^6^ tumor cell lysate-pulsed BMDCs or CurDCs. Some mice were given IL-33 every 3 days, beginning on the day of the first immunization. Mice that received PBS served as controls. Shown are the tumor growth curves. The experiments were performed twice with a total of 10 mice per group (*n* = 10). Results shown are the mean ± SD of two combined experiments. **P < 0.01.

To examine the role of IL-33 in the antitumor efficacy induced by dectin-1-activated DCs, B16-OVA melanoma tumor and OT-II transgenic mouse models were used. B16-OVA tumor-bearing OT-II mice were immunized with OT-II OVA peptide-pulsed BMDCs or CurDCs with or without addition of IL-33. CurDCs plus IL-33 induced more potent inhibition on melanoma tumor growth than CurDCs alone (Figure 4A), while IL-33 had no effects on melanoma tumor growth inhibition in BMDC-treated mice (Figure 4A). OT-II transgenic mice are an artificial mouse model and OT-II T cells target a single artificial antigen. To further verify the capacity of IL-33 in dectin-1-activated DC-induced antitumor efficacy, MPC-11 myeloma mouse model was used. Similarly, immunization with CurDCs plus IL-33 significantly slowed MPC-11 tumor growth as compared to CurDC immunization (Figure 4B), whereas IL-33 had marginal effects on BMDC-induced antitumor efficacy (Figure 4B). These results indicated that IL-33 promoted dectin-1-activated DC- but not BMDC-induced antitumor immunity *in vivo*.

### Dectin-1-Activated DCs Plus IL-33 Fail to Support Treg Cell Differentiation

**Figure 5:**
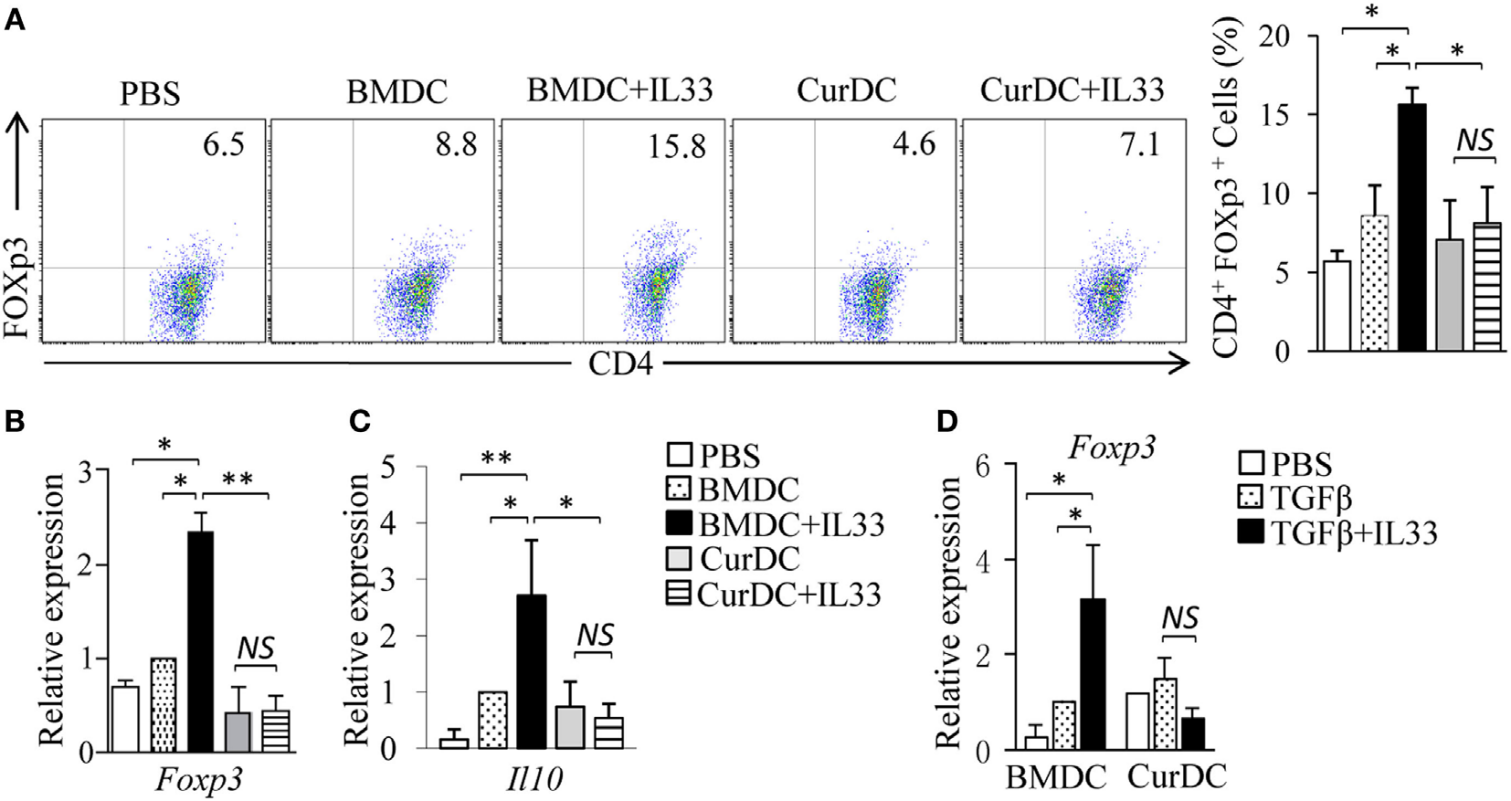
The role of interleukin (IL)-33 in dendritic cell (DC)-induced regulatory T (Treg) differentiation. **(A–C)** OT-II mice (two to three mice/group) were immunized twice (1 week apart) with OVA-peptide-pulsed BMDCs or CurDCs. In addition, some of the mice immunized with BMDCs or CurDCs were given IL-33 (250 ng/mouse) every 3 days, beginning on the day of the first immunization. Mice received PBS served as controls. Mouse spleen cells were restimulated with BMDCs or CurDCs in the presence or absence of IL-33 (50 ng/mL) for 2 days in the culture. **(A)** Flow cytometry analysis of Foxp3^+^CD4^+^ T cells. Right, summarized results of three independent experiments obtained as at left. The experiments were performed three times (*n* = 3). **(B,C)** Quantitative polymerase chain reaction (qPCR) examined the expression of *Foxp3* **(B)** and *Il10* **(C)** in CD4^+^ T cells. The experiments were performed three times (*n* = 3). **(D)** Naïve CD4^+^ T cells were cocultured with BMDCs or CurDCs under Treg [transforming growth factor β (TGFβ)] polarizing conditions in the presence (TGFβ + IL33) or absence of IL-33. qPCR examined the expression of *Foxp3* in CD4^+^ T cells. The experiments were performed three times (*n* = 3). Data are representative of three **(A)** independent experiments or presented as mean ± SD of three **(A–D)** independent experiments. *NS*, non-significant; *P < 0.05; **P < 0.01.

Interleukin-33 was shown to promote Treg cell development (34, 35). To explore the effects of IL-33 on Treg cell differentiation in DC-immunized mice, OT-II mice were immunized with OVA peptide-pulsed BMDCs or CurDCs with or without the addition of IL-33. Indeed, mice immunized with BMDCs plus IL-33 displayed significantly higher production of Foxp3^+^CD4^+^ Treg cells (Figure 5A) and expression levels of *Foxp3* and *Il10* in T cells (Figures 5B,C) than mice receiving CurDCs, CurDCs plus IL-33, BMDCs, or PBS control. However, there was no increase of Foxp3^+^CD4^+^ Treg cells (Figure 5A) and *Foxp3* and *Il10* gene expression in T cells (Figures 5B,C) from mice immunized with CurDCs plus IL-33 compared to CurDCs alone. We next examined the effects of IL-33 on DC-induced Treg cell differentiation *in vitro*. Similarly, the addition of IL-33 increased *Foxp3* expression in BMDC-treated Treg cells (Figure 5D), but not in CurDC-treated cells (Figure 5D). Together, these results demonstrated that the combination of dectin-1-activated DCs and IL-33 fails to support Treg cell differentiation.

### IL-33 Promotes the Proliferation of Dectin-1-Activated DC-Treated Th Cells

**Figure 6:**
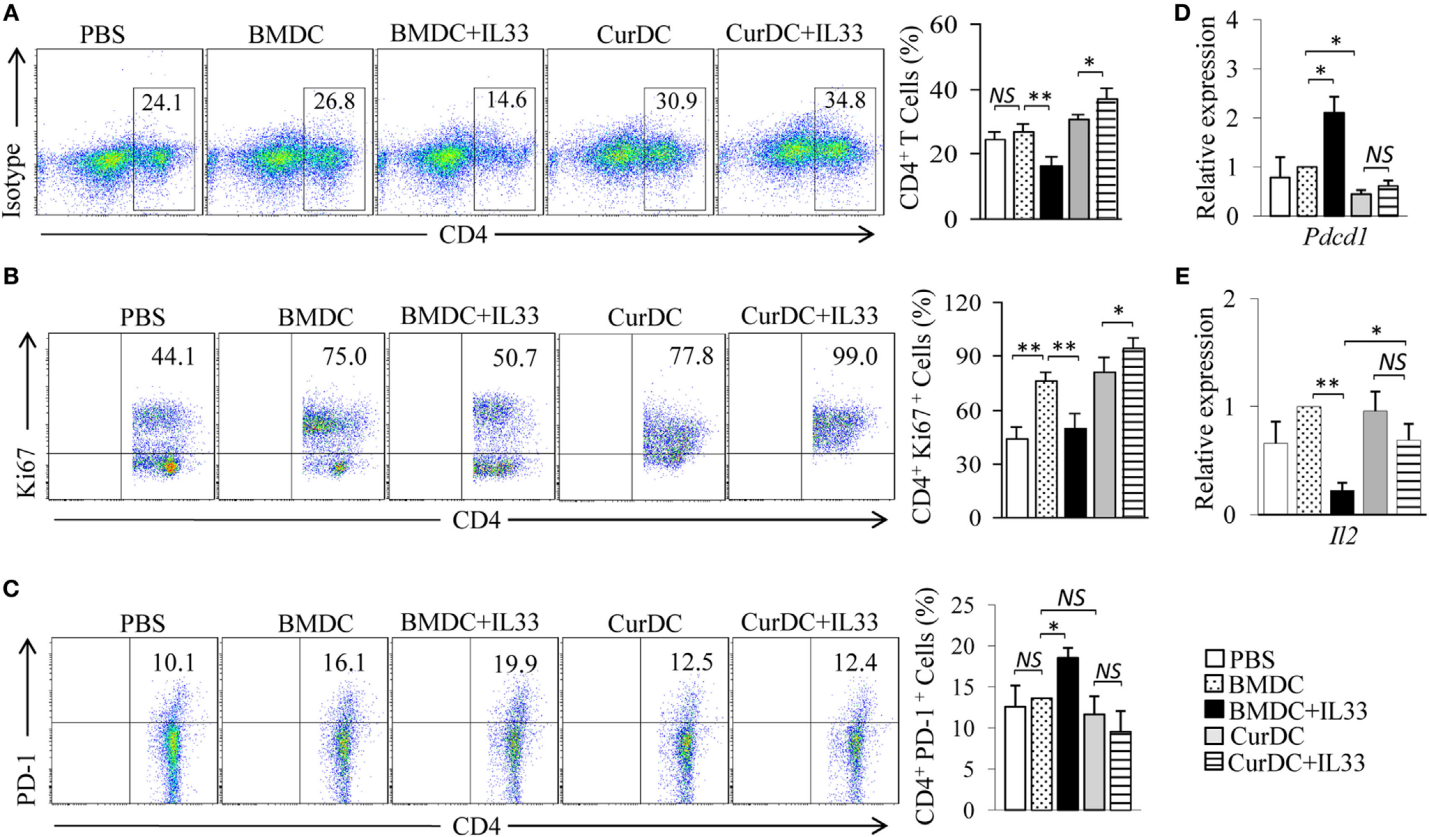
Interleukin (IL)-33 maintains the cell proliferation capability of dectin-1-activated dendritic cell (DC)-primed T helper cells *in vivo*. OT-II mice (two to three mice/group) were immunized twice (1 week apart) with OVA-peptide-pulsed BMDCs or CurDCs. In addition, some of the mice immunized with BMDCs or CurDCs were given IL-33 (250 ng/mouse) every 3 days, beginning on the day of the first immunization. Mice received PBS served as controls. Mouse spleen cells were restimulated with BMDCs or CurDCs in the presence or absence of IL-33 (50 ng/mL) for 2 days in the culture. **(A)** Flow cytometry analysis of CD4^+^ T cells in mouse spleen cells. Right, summarized results of three independent experiments obtained as at left. The experiments were performed three times (*n* = 3). **(B)** Flow cytometry analysis of Ki67^+^CD4^+^ T cells. Right, summarized results of three independent experiments obtained as at left. The experiments were performed three times (*n* = 3). **(C)** Flow cytometry analysis of PD-1^+^CD4^+^ T cells. Right, summarized results of three independent experiments obtained as at left. The experiments were performed three times (*n* = 3). **(D,E)** CD4^+^ T cells were isolated by the magnetic cell sorting. Quantitative polymerase chain reaction examined the expression of *Pdcd1* **(D)** and *Il2* **(E)** in CD4^+^ T cells. The experiments were performed three times (*n* = 3). Data are presented as mean ± SD of three **(D,E)** independent experiments. *NS*, non-significant; *P < 0.05; **P < 0.01.

The expansion of antitumor effector T cells is critical for antitumor immunity *in vivo*. OT-II mice were used and the effects of dectin-1-activated DCs and IL-33 on the expansion of CD4^+^ T cells were examined. As shown in Figure 6A, mice immunized by CurDCs compared to BMDCs had comparable frequencies of CD4^+^ T cells in spleen cells. Interestingly, the addition of IL-33 increased the CD4^+^ T cell frequencies in CurDC-immunized mice (Figure 6A) but reduced the frequencies in BMDC-immunized mice (Figure 6A). To determine the effects of dectin-1-activated DCs and IL-33 on Th cell proliferation, T cells from spleen cells and tumor tissues were analyzed by intracellular staining of Ki67. IL-33 increased Ki67 expression in CD4^+^ T cells from CurDC-treated mice (Figure 6B; Figures S6C,D in Supplementary Material), but decreased its expression in CD4^+^ T cells from BMDC-treated mice (Figure 6B; Figures S6C,D in Supplementary Material). These results demonstrated that the combination of IL-33 with CurDCs but not BMDCs promoted the proliferation of CD4^+^ T cells *in vivo*.

We next examined the phenotype of Th cells from mice immunized by DCs plus IL-33. CurDCs slightly inhibited Th cell expression of PD-1 as compared to BMDCs (Figures 6C,D; Figure S6E in Supplementary Material). Though IL-33 had minor effects on Th cell expression of PD-1 in CurDC-immunized mice (Figures 6C,D; Figure S6E in Supplementary Material), IL-33 potently increased Th cell expression of PD-1 in BMDC-immunized mice (Figures 6C,D; Figure S6E in Supplementary Material). Furthermore, IL-33 inhibited *Il2* expression in Th cells from mice immunized by BMDCs but not CurDCs (Figure 6E; Figure S6F in Supplementary Material). These results indicated that IL-33 promoted an exhaustive phenotype of Th cells in mice immunized with BMDCs but not CurDCs, suggesting the potential mechanism for the high proliferation capability of Th cells in mice immunized by CurDCs plus IL-33.

## Discussion

Tumor-specific Th9 cells are potent antitumor effector cells (10, 11). We recently reported that dectin-1-activated DCs promote Th9 cell differentiation and trigger potent therapeutic effects against established tumors, better than regular BMDCs (12, 13). However, mechanisms underlying the induction of Th9 cells and antitumor efficacy by dectin-1-activated DCs are not fully defined. IL-33 is one of the 42 upregulated genes of cytokines and costimulatory molecules in dectin-1-activated DCs (12). And we also confirmed the upregulation of IL-33 expression in dectin-1-activated DCs in this study. However, the role of IL-33 in the induction of Th9 cells and antitumor immunity by dectin-1-activated DCs still remains unknown. IL-33 is a nuclear cytokine, which is released *via* cell necrosis (36). Matured DCs will undergo cell death *in vitro* and *in vivo* and IL-33 can be released from these dying dectin-1-activated DCs, which may affect T cell differentiation primed by dectin-1-activated DCs. Indeed, in this study, we found that blocking IL-33/ST2 axis inhibits dectin-1-activated DC-induced Th9 differentiation *in vitro* and *in vivo*. In addition, the addition of IL-33 further promotes the development of Th9 and Th1 cells induced by dectin-1-activated DCs. More importantly, the addition of IL-33 further increases dectin-1-activated DC-induced antitumor efficacy in mouse models. Consistently, a recent report showed that IL-33-treated IL-9-producing T cells have higher antileukemic capabilities than untreated cells (32). Thus, our data demonstrate the important role of IL-33 in dectin-1-activated DC-induced Th9 cell differentiation and antitumor efficacy.

Cytokine milieu is the major determinant for Th cell differentiation (5, 37). The cytokine IL-33 itself may promote the differentiation of Th1 or Th9 cells, depending on the cytokine milieu (24, 32). Regular matured DCs express high levels of IL-12, a key cytokine for Th1 cell differentiation (38), which along with IL-33 directs naïve CD4^+^ T cells to differentiate into Th1 cells. However, dectin-1-activated DCs produce large amounts of TNF superfamily cytokines, such as TNFSF15 and OX40L (12, 14, 15), which along with IL-33 may further promote Th9 cell formation. Th1 cells are terminally differentiated, shorter lived effector cells (39, 40), however, Th9-derived IL-9 has the potential to enhance the survival and proliferation of T cells (12). In addition, IL-33 itself may stimulate the survival and proliferation of T cells (41). Thus, these observations may account for the capability of IL-33 in promoting Th9 cell production and antitumor immunity in dectin-1-activated DC-immunized mice but not in BMDC-immunized mice.

In this study, we found that IL-33 increases the production of Foxp3^+^ Treg cells in mice immunized by BMDCs but not dectin-1-activated DCs. Treg cells can be divided into naturally occurring (nTreg) and inducible (iTreg) regulatory cells (42). nTreg cells are generated in the thymus and are 5–10% of the total peripheral CD4^+^ T cells (43). By contrast, iTregs are peripherally induced from naive CD4^+^ T cells in response to foreign antigens in the presence of TGF-β1 (44, 45). These observations suggest that IL-33 increases the production of iTreg but not nTreg cells in BMDC-immunized mice. It remains unclear about mechanisms of the different function roles of IL-33 in Treg cell differentiation in BMDC- and dectin-1-activated DC-immunized mice. But dectin-1-activated DCs overexpress the TNF family cytokines TNF-α, TNFSF15 (TL1A), OX40L, and TNFSF8 (12, 13). Among these cytokines, OX40L inhibits Treg cell differentiation and TL1A inhibits the immunosuppressive function of Treg cells (46–49). Based on these observations, we speculate that the specific profile of cytokines and costimulatory molecules expressed by dectin-1-activated DCs, especially OX40L and TL1A may contribute to the inhibition of Treg cell differentiation induced by IL-33.

In conclusion, our study demonstrates that IL-33 contributes to the induction of Th9 and Th1 cells by dectin-1-activated DCs. The addition of IL-33 further promotes dectin-1-activated DC-induced antitumor efficacy. The combined use of dectin-1-activated DCs and IL-33 may present a new effective modality of DC-based vaccines in tumor immunotherapy. Our study may have important clinical implications.

## Ethics Statement

This study was carried out in accordance with the recommendations of “the ethical guidelines of the Animal Ethical Committee of First Hospital of Jilin University.” The protocol was approved by the “Animal Ethical Committee of First Hospital of Jilin University.”

## Author Contributions

SW initiated the study; SW designed the experiments and wrote the paper; SW, JC, and YZ performed majority of the experiments; YJ, YW, DW, and AW performed some experiments; AW read and edited the manuscript; QY, SG, HY, and RG provided critical suggestions to this study.

## Conflict of Interest Statement

The authors declare that the research was conducted in the absence of any commercial or financial relationships that could be construed as a potential conflict of interest.
