# Supplementary material for: Interleukin-33 Contributes to the Induction of Th9 Cells and Antitumor Efficacy by Dectin-1-Activated Dendritic Cells
Source: Front Immunol. 2018 Jul 31;9:1787. doi: 10.3389/fimmu.2018.01787 (PMC6079242; doi:10.3389/fimmu.2018.01787)
Supplement: Supplementary file 2 [file Data_Sheet_1.PDF]

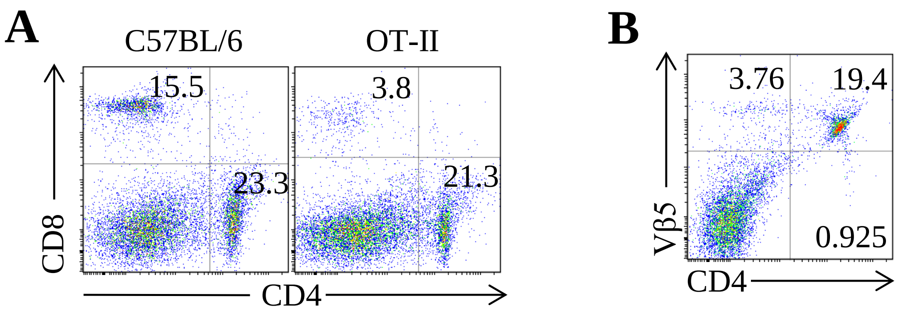

Figure S1. Phenotypic analysis of OT-II transgenic mice. (**A**) Flow cytometry analysis of $CD4^+$ and $CD8^+$ T cells in spleen cells collected from C57BL/6 and OT-II mice. Numbers in the dot plots represent the percentages of $CD4^+$ or $CD8^+$ cells. (**B**) Flow cytometry analysis of Vβ5-expressing $CD4^+$ T cells in spleen cells collected from OT-II mice. Numbers in the dot plots represent the percentages of $V\beta5^+CD4^-$, $V\beta5^-CD4^+$ or $V\beta5^+CD4^+$ cells. Data are representative of three independent experiments .

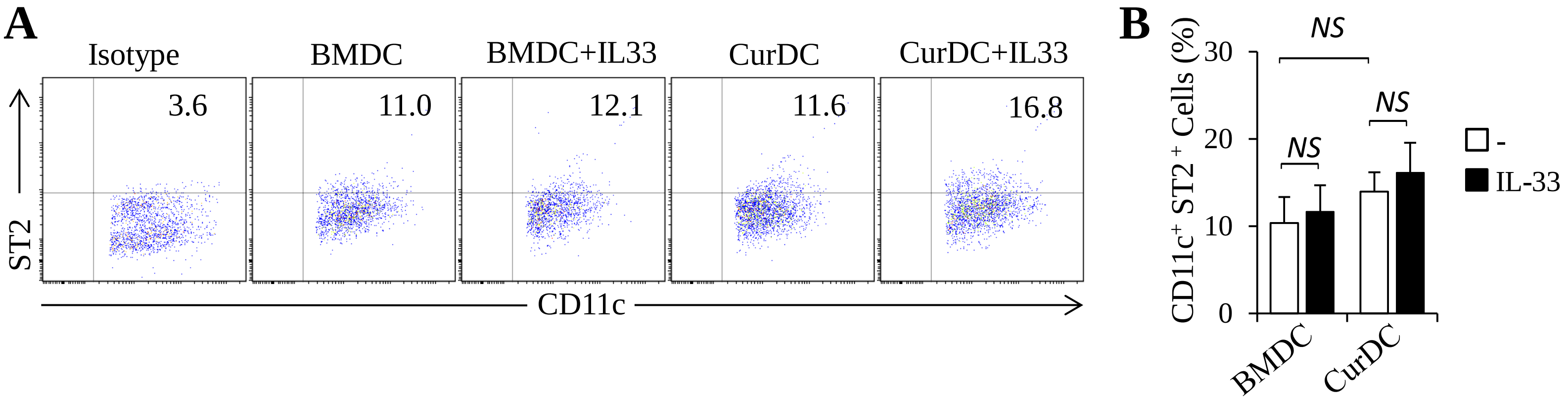

Figure S2. The expression of ST2 in BMDCs and CurDCs with or without IL-33 treatment. Mouse iDCs were matured by TNF-α/IL-1β (BMDC), Curdlan (CurDC) in the presence of absence of IL-33 (50 ng/mL) for 48 hours. (**A**) flow cytometry analysis of $ST2^+CD11c^+$ cells. Numbers in the dot plots represent the percentages of $ST2^+CD11c^+$ cells. (**B**) summarized results of three independent experiments obtained in (A). The experiments were performed three times (n=3). Data are representative of three (A) independent experiments or presented as mean ± SD of three (B) independent experiments . *NS*, non-significant.

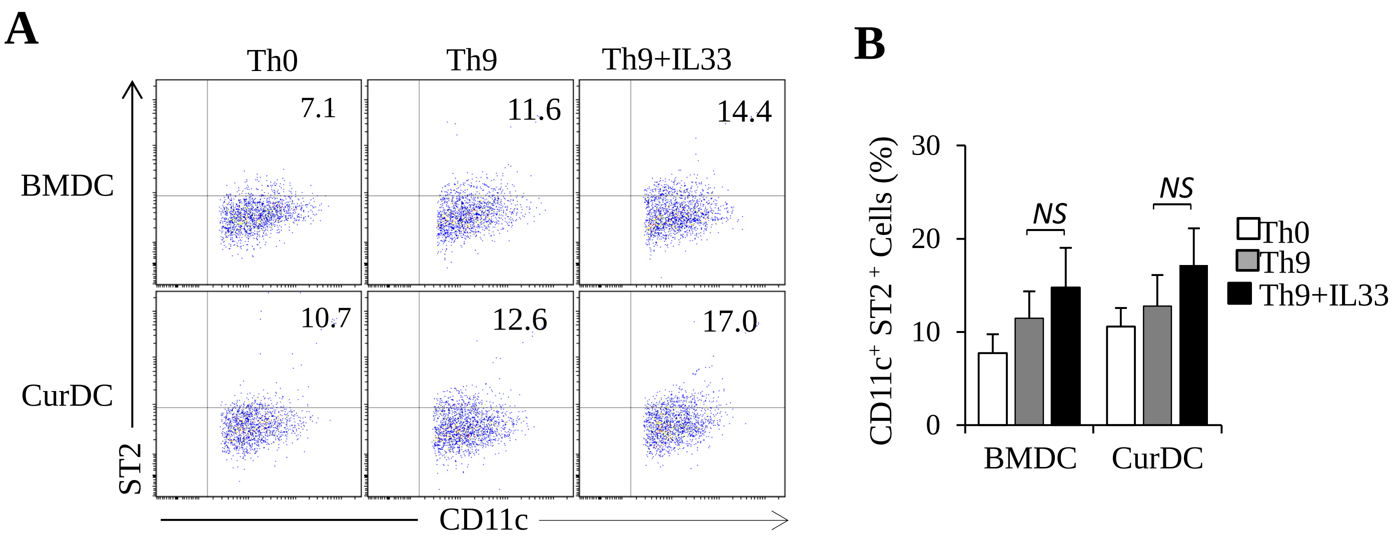

Figure S3. The effects of IL-33 on ST2 expression by BMDCs and CurDCs in the *in vitro* Th9-priming cultures. Naïve $CD4^+$ T cells were cocultured with BMDCs or CurDCs under Th9 polarizing conditions in the presence or absence of IL-33. Cell cultures without (Th0) addition of Th9-polarizing cytokines TGF-β and IL-4 were used as controls. Cells were cultured for 24 hours. (**A**) flow cytometry analysis of $ST2^+CD11c^+$ cells. Numbers in the dot plots represent the percentages of $ST2^+CD11c^+$ cells. (**B**) summarized results of three independent experiments obtained in (A). The experiments were performed three times (n=3). Data are representative of three (A) independent experiments or presented as mean ± SD of three (B) independent experiments . *NS*, non-significant.

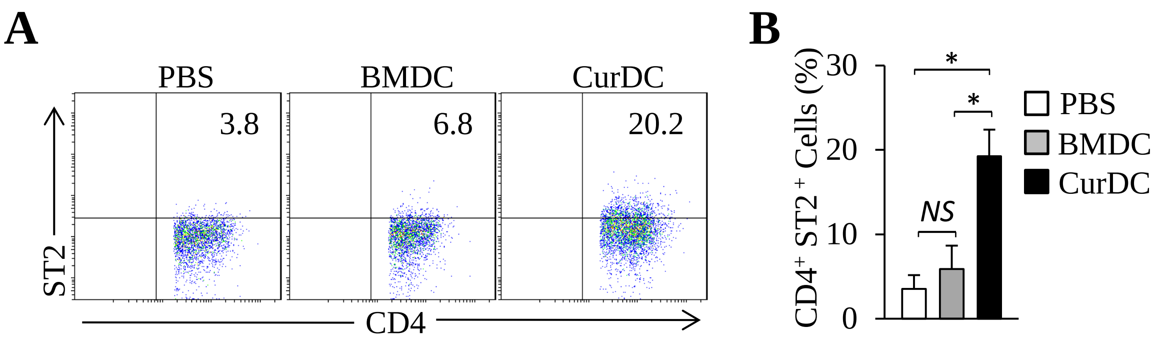

Figure S4. Dectin-1-activated DC immunization stimulates ST2 expression by CD4$^+$ T cells. C57BL/6 mice (2 to 3 mice/group) were immunized twice (one week apart) with TRP1-peptide-pulsed BMDCs or CurDCs. PBS served as control. On day 3 after the 2nd immunization, total leukocytes from spleen cells were harvested and restimulated with TRP1-peptide-pulsed BMDCs and CurDCs respectively for 48 hours in the culture. Cells from PBS control mice were cultured without addition of DCs. (**A**) flow cytometry analysis of ST2$^+$CD4$^+$ T cells. Numbers in the dot plots represent the percentages of ST2$^+$CD4$^+$ T cells. (**B**) summarized results of three independent experiments obtained in (A). The experiments were performed three times (n=3). Data are representative of three (A) independent experiments or presented as mean ± SD of three (B) independent experiments . *NS*, non-significant; **P < 0.05* (Student *t* test).

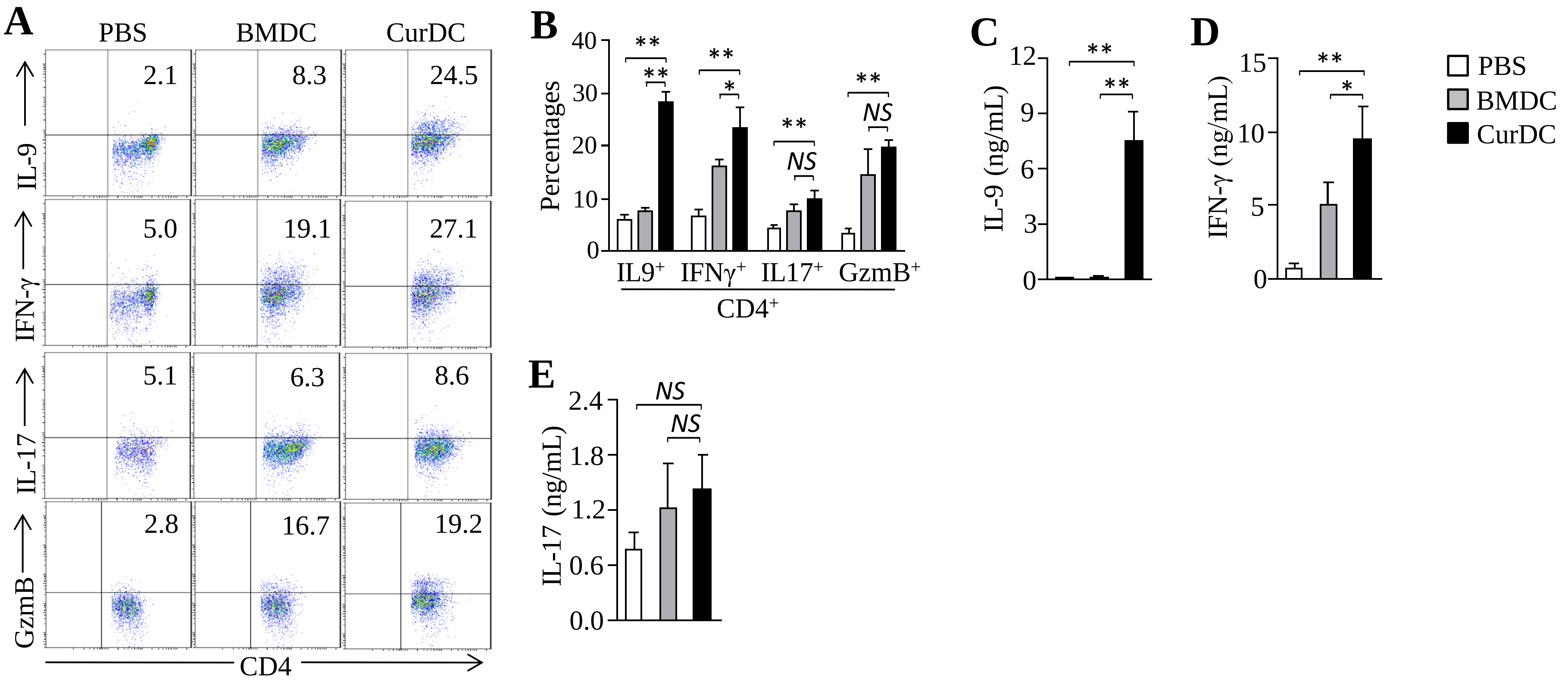

Figure S5. Antitumor effector cells induced by dectin1-DC in vivo. OT-II mice (2 to 3 mice/group) were immunized twice (one week apart) with OVA-peptide-pulsed BMDCs or CurDCs. PBS served as control. On day 3 after the 2nd immunization, total leukocytes from spleen cells were harvested and restimulated with OVA-peptide-pulsed BMDCs and CurDCs respectively for 48 hours in the culture. Leukocytes from PBS control mice were cultured without addition of DCs. (**A**) Flow cytometry assessed the percentages of IFN-γ-, IL-9-, IL-17-,or GzmB-producing $CD4^+$ T cells. Numbers in the dot plots represent the percentages of Th cells. (**B**) Summarized results of three independent experiments obtained in (A). The experiments were performed three times (n=3). (**C-E**) ELISA examined the protein levels of IL-9 (**C**), IFN-γ(**D**) and IL-17 (**E**) in the culture supernatants. The experiments were performed three times (n=3). Data are representative of three (A) independent experiments or presented as mean ± SD of three (B-E) independent experiments . *NS*, non-significant; *$*P < 0.05$; $**P < 0.01$*(Student *t* test).

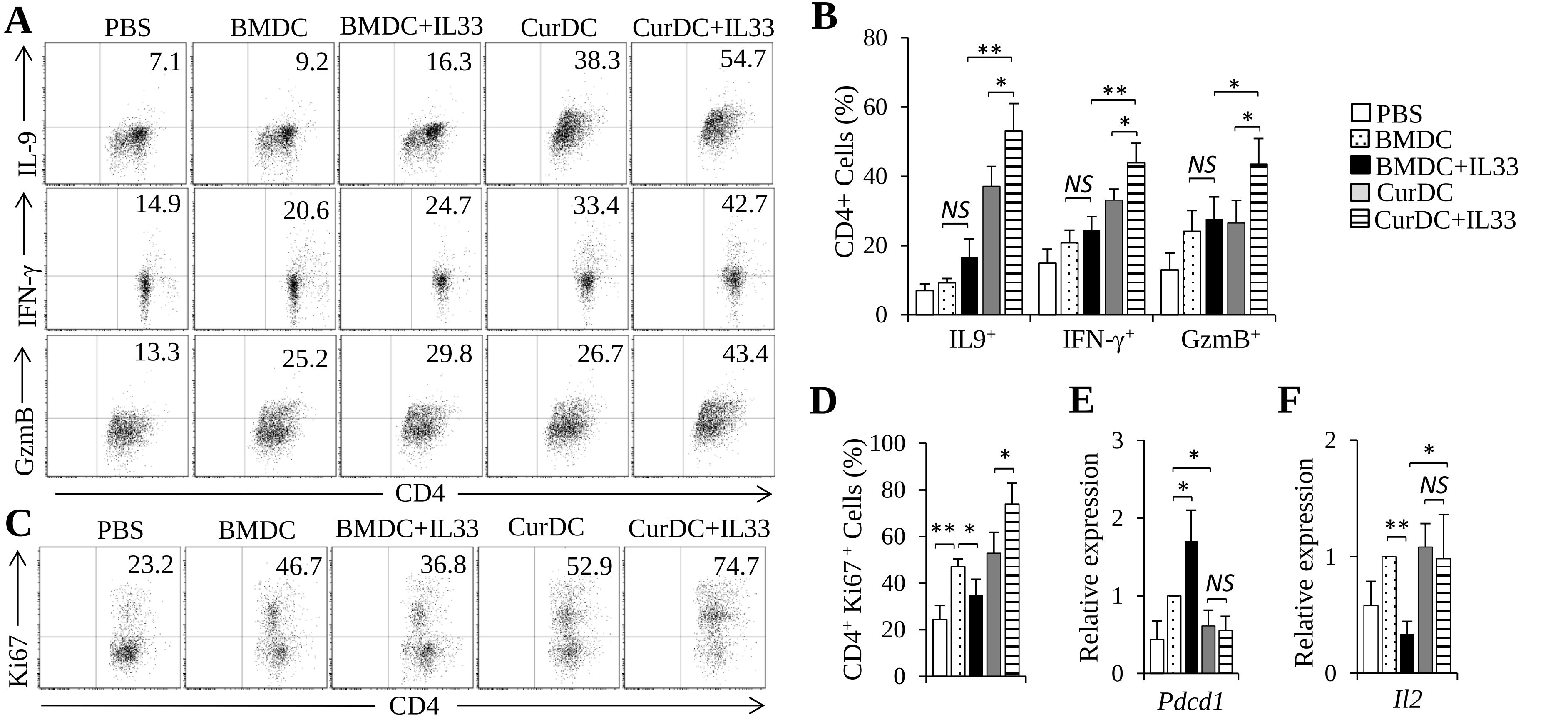

Figure S6. The analysis of tumor infiltrating T cells in mice immunized with dectin-1-activated DCs plus IL-33. OT-II mice were injected subcutaneously with 1×10^5 B16-OVA cells. On day 3 after tumor challenge, mice (2 to 3 mice/group) were given two weekly subcutaneous immunizations with 1×10^6 OVA-peptide-pulsed BMDCs or CurDCs with or without addition of IL-33 (250 ng/mouse). On day 3 after the second immunization, CD4$^+$ T cells were isolated from tumor tissues by MACS and restimulated with BMDCs or CurDCs in the presence or absence of IL-33 (50 ng/mL) respectively for 24 hours. (**A**) Flow cytometry analysis of IL-9- IFN-γ or GzmB-producing CD4$^+$ T cells. Numbers in the dot plots represent the percentages of double-positive T cells. (**B**) summarized results of three independent experiments obtained in (A). The experiments were performed three times (n=3). (**C**) Flow cytometry analysis of Ki67$^+$CD4$^+$ T cells. Numbers in the dot plots represent the percentages of Ki67$^+$CD4$^+$ T cells. (**D**) summarized results of three independent experiments obtained in (C). The experiments were performed three times (n=3). (E,F) CD4$^+$ T cells were isolated by MACS. qPCR examined the expression of *Pdcd1* (**E**) and *Il2* (**F**) in CD4$^+$ T cells. The experiments were performed three times (n=3). Data are representative of three (A,C) independent experiments or presented as mean ± SD of three (B,D-F) independent experiments . *NS*, non-significant; **P* < 0.05; ***P* < 0.01.
